# Achieving outstanding mechanical/bonding performances by epoxy nanocomposite as concrete–steel rebar adhesive using silane modification of nano SiO_2_

**DOI:** 10.1038/s41598-023-36462-0

**Published:** 2023-06-06

**Authors:** Reza Ghamarpoor, Masoud Jamshidi, Majid Mohammadpour

**Affiliations:** Constructional Polymers and Composites Research Lab., School of Chemical, Petroleum and Gas Engineering, Iran University of Science and Technology (IUST), Tehran, Iran

**Keywords:** Engineering, Materials science, Nanoscience and technology

## Abstract

Anchoring steel rebar in concrete structures is a common method in the building and construction industry. This research focuses on improving the mechanical/bonding properties of the prepared epoxy nanocomposite adhesive using surface treatment of SiO_2_ nano fillers by glycidoxypropyltrimethoxysilane (GPTMS). For this purpose, the nano silica particles were silanized via a facile sol–gel method at silane concentrations of 1, 5, 10 and 20X (i.e. X is stoichiometric silane concentration). The nanoparticles were characterized carefully by FTIR, TGA, XRD and XPS techniques. It was found that the highest GPTMS grafting ratio was obtained at silane concentration of 10X. The pure and silanized nanoparticles were added to a two-pack epoxy resin and were compared for tensile and compressive properties. It was found that surface modification of nano silica caused improvement in the strength, modulus, compressive strength and compressive modulus by 56, 81, 200 and 66% compared to the pristine epoxy adhesive and also 70, 20, 17 and 21% compared to the pure nano silica containing adhesive. It also caused 40 and 25% improvement in the pullout strength, 33 and 18% enhancement in the pullout displacement and 130 and 50% in adhesion energy compared to the pristine and raw silica-containing adhesives, respectively.

## Introduction

Concrete is one of the most valuable building materials that is found wherever there is an infrastructure^1–4^. Rebars, in the form of reinforcement, are used to network the foundation and columns^5–7^. Also, another one of their most important applications in the foundation is the implementation of rabbits to prevent construction joints in concrete^8^. To reinforce the concrete in the structures, the rebars are connected with wire, so that the anchor system is firmly placed in the concrete^9,10^.

The bond between cement and steel rebar plays an important role in the physicochemical behavior of the anchor system^10,11^. In general, cracks in concrete are engendered by tensile stresses, which can be resulted by over load, temperature changes, and shrinkage, and resulted in poor connections, rebar slippage, and anchor failure^12,13^. Many efforts have been performed to improve the adhesion of steel rebar in cement concrete, including application of polymeric materials on the rebar surface (e.g. coatings^14^, epoxy resins^15^, etc.) and modifying the concrete mix design (e.g. with nanoparticles^16^, fibers^17^, etc.).

Generally concrete adhesives are divided into two types: epoxy based and latex/polymer based^18^. This kind of materials are used to repair concrete, seal concrete, increase the physicochemical properties of concrete, and connect old concrete to new one^19^. Some of the advantages of structural adhesives are strengthening the structure and augmenting the connection between materials, using in wet environments, connecting members with a cross-section, corrosion resistance and quick and easy implementation^20,21^. Planting rebar or bolt is one of the most commonly used methods in the construction industry. This includes a wide range of structural and non-structural connections as well as strengthening of the structures^22,23^. From this starting point, improving adhesive properties is the most efficient technique to prevent structural damages using strengthening the metal-to-concrete bonding^24^.

Nowadays, epoxy-based adhesives are of the most popular adhesives for steel rebar anchoring systems, due to their high adhesion strengths, fast and excessive compaction, impermeability against moisture, seawater, sewage and petroleum materials, significant resistance to vibrations and structural stresses and high mechanical properties^19,25^.

From 1980 onwards, researchers such as Bloxham^26^, Van Gemert et al.^27^ and Swamy et al.^28^ conducted studies on the reinforcement of concrete with steel rebar using epoxy adhesives.

Many scientists have investigated the mechanical and chemical behavior between adhesive bars and concrete^29,30^. It has been determined that the rebar with a larger diameter has higher failure in the connection with concrete, which in order to have a better connection and no brittleness, the length of the rebar should be fifteen times the diameter of the rebar^31,32^.

Zhao et al.^33^ investigated the bonding of steel rebar to concrete structure using epoxy adhesive. It was reported that the adhesive had good bond strength to both rebar and concrete and the failure of the system occurred at the interfaces of the adhesive, but the ductility of anchoring system decreased using epoxy adhesive.

In recent years, fillers are used to improve the physical and mechanical properties of adhesives^34–38^. It has been shown that the type, size and properties of fillers have a great impact on the bonding strength of adhesive joints and stress transfer between concrete and steel rebar^39^. Szymanowski et al.^40^ investigated the effect of using tetragonal crystalline titanium oxide nanoparticles (TiO_2_) in adhesive and its effect on bonding strength in layered cementitious composite. The results showed that inclusion of 0.5 wt% of TiO_2_ nanoparticles to the adhesive increased its bonding properties, wear resistance, tensile strength and hardness. Ismael et al.^41^ used nano SiO_2_ and AL_2_O_3_ in cementitious composites reinforced by steel fibers. The results showed that the addition of nanoparticles led to an increase in the bond between steel and matrix especially at higher cement contents. Using Al_2_O_3_ nanoparticles also caused decline in cracking. May et al.^42^ used multiwall carbon nanotubes (MWCNTs) to reinforce diglycidyl ether epoxy resin by sol–gel method. The results showed that the presence of nanoparticles causes better adhesion properties and tensile strength (up to 28.5 MPa) of epoxy resin. Li et al.^43^ modified silica nanoparticles with polymethylhydrosiloxane (PMHS) through the sol–gel process and used it for surface modification of materials. The results showed that the created interfacial chemical bonds enhanced hydrophobicity of the surface. It was found that fillers and chemical coupling agents could strengthen the connection between concrete and steel rebar. The effect of micro and nano silica particles on epoxy adhesives was also investigated. It was found that silica micro particles and their particle size affects the mechanical properties of epoxy adhesive and bonding strength to steel bar. However, silica nanoparticles caused decline in the properties that this was attributed to the tendency of hydrophilic nanoparticles to agglomeration in organic matrix^44^.

Based on the literature review, it was found that using of nanoparticles in anchoring adhesive has been studied in few researches. Furthermore, in most cases just the bond strength between concrete and rebar has been investigated and the effects on the mechanical properties of adhesive and its relation to concrete-rebar adhesion have not been assessed.

On this basis, the silica nanoparticles was selected as cost-effective filler in this research. It was surface modified by sol–gel method using an epoxy based silane coupling agent at different concentrations. The silane grafting was assessed by FTIR, TGA, XRD, SEM and XPS analysis. The raw and surface modified fumed silica were applied to a two pack epoxy adhesive. The prepared pure and modified nanocomposite adhesives were evaluated for flexural and compressive properties. The best adhesives (based on the mechanical properties) were used for anchoring of steel rebar in concrete. FE-SEM Analysis was used for characterization of the fractured surfaces of the adhesive samples. Finally, the effects of pure and modified nanoparticles on the concrete-rebar adhesion performances were studied.

## Experimental

### Material

Nanya NPEL-128 epoxy resin (Taiwan) and Epikure F205 as curing agent were used as binder in this study. Fumed silica nanoparticles with an average primary particle size of 25–35 nm was used as reinforcing nano filler for epoxy adhesives. Glycidoxypropyltrimethoxysilane (GPTMS) (GLYMO, Evonic Company) was used surface modification of fumed silica. Absolute ethanol (99.98%, Merck, Germany) was used as solvent.

### Methods

#### Surface treatment of fumed silica

Firstly, the optimal amount of silane required to modify the surface of nanoparticles was calculated by the following stoichiometric relationship^45–48^:

1 $$ m_{GPTMS} = 6\frac{{M_{GPTMS} \cdot m_{sio2} \cdot n_{OH} \cdot S_{sio2} \cdot 10^{18} }}{{N_{A} }} $$

where m_GPTMS_ and M_GPTMS_ are the mass of the GPTMS (gr) and the molecular mass of the GPTMS, respectively. N_A_ and n_OH_ are Avogadro numbers and the number of hydroxyl groups, respectively.

This value varied depending on the molecular mass of the GPTMS, the specific surface area (BET) of the fumed nanoparticles and the TGA data (see Section 1S in supporting information).

**Scheme 1.**
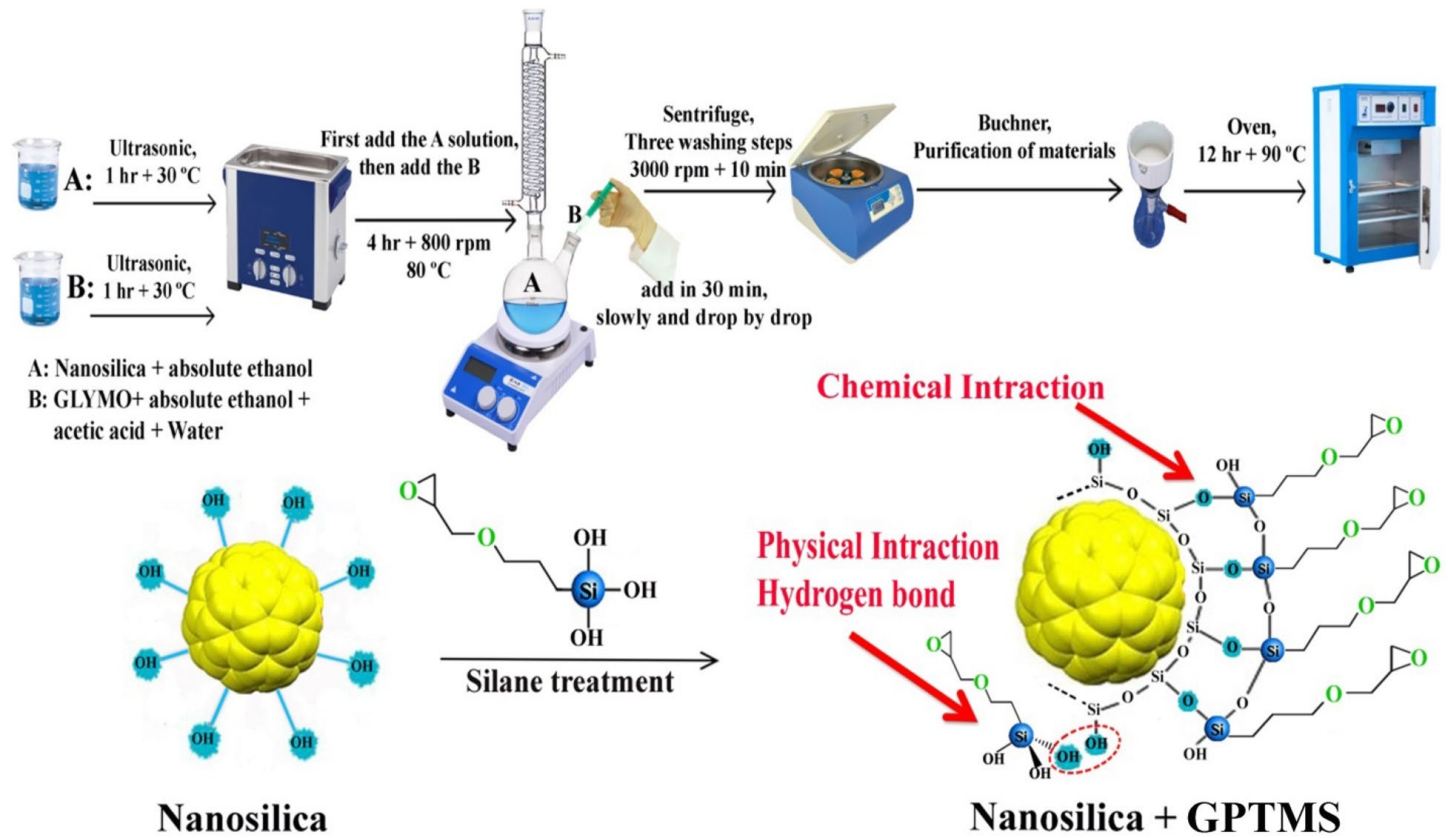
Surface treatment process of fumed silica by GPTMS.

Scheme 1 represents the surface treatment process of fumed silica by GPTMS. For this purpose, 0.5 g of fumed silica were added to 70 g of ethanol and sonicated at 30 °C for 1 h. Afterward, the GPTMS (i.e. at the content that calculated by Eq. 1) was added to ethanol, water and acetic acid (at a weight ratio of 0.1:0.05) to hydrolyze the GPTMS. The optimal pH of the hydrolysis solution was determined by zeta potential analysis. At this point, the prepared suspension of fumed silica in ethanol was poured into a flask. The solution was stirred and added to the reactor dropwise during 30 min. Thereafter the solution was mixed for 4 h and centrifuged. The sediments were separated and washed three times with acetone to remove unreacted GPTMS molecules. Finally, the modified fumed silica were dried in an oven for 12 h at 90 °C for GPTMS condensation on the nanoparticles surface.

To enhance grafting ratio, the nanoparticles were also modified at silane concentrations of 5X, 10X and 20X (see Table 1).

**Table 1:** The ingredients for preparation of modified nanoparticles.

Samples	Nanoparticle suspension		Silane solution			
	Nano silica (g)	Absolute ethanol (ml)	GPTMS (ml)	Acetic acid (ml)	Absolute ethanol (ml)	Water (ml)
NS	0.5	–	–	–	–	–
NS-G-X	0.5	70	0.657	0.05	25	0.1
NS-G-5X	0.5	70	3.285	0.05	25	0.1
NS-G-10X	0.5	70	6.57	0.05	25	0.1
NS-G-20X	0.5	70	13.14	0.05	25	0.1

#### Preparing nanocomposite adhesives

To prepare pristine epoxy adhesive, the hardener was added to resin part at weight ratio of 1:2. The mixture was gently stirred to inhibit air bubble creation.

To prepare nanocomposite samples, nanoparticle (i.e. the raw or modified fumed silica) was added to the resin part (at 0.5, 1, 3 and 5 wt%). For this purpose, the fumed silica were added to n-butanol and sonicated at 28 °C and 200 W (frequency of 40 kHz) for 1 h. The resulted suspension was added gently to the resin and mixed slowly at 350 rpm for 30 min. The suspension was applied gently to the resin and mixed slowly at 450 rpm and put in a vacuum oven to remove the solvent. To prepare pure and modifies epoxy adhesive samples (see Table 2), the curing agent was applied to the resins based part at weight ratio of 1:2 and mixed gently^49^, and placed in ambient conditions for 10 h. Thereafter, the solidified specimens were removed and placed in an oven at 100 °C for 5 h for post curing.

**Table 2:** The prepared adhesives.

Samples	Codes	Nano silica (wt%)
Pristine adhesive*	Ad-Ctrl	–
Raw fumed silica containing adhesive	Ad-NS	0.5, 1, 3, 5
Modified fumed silica nano (MNS) containing adhesive	Ad-MNS	1

*Epoxy resin + curing agent.

#### Preparing concrete samples

The purpose of this study is to prepare an epoxy adhesive for effective anchoring of steel rebar in concrete. For this purpose, the concrete at the water-to-cement (w/c) ratio of 1:1.89 was prepared (see Table 3). The prepared concrete was poured into the cylindrical molds (see Sect. 2S in supporting information). The plastic bar (1 cm in diameter and 10 cm in length) was used to form anchoring hole in the concrete without drilling. The filled molds were held in ambient conditions for 24 h. Thereafter, the samples were placed in the water for 28 days to be cured. After curing of the concrete specimens, the steel rebar (0.8 cm in diameter and 20 cm in length) was inserted in the center of the hole (with 10 cm length) and then adhesive was inserted into the gap (i.e. 0.2 cm thickness) between the steel rebar and concrete wall. The specimens were placed at ambient temperature for 14 days to be cured.

**Table 3:** Materials used in concrete.

Components	Content (kg/m^3^)
Cement	305
Sand	615
Coarse aggregate	310
Water	161
Super plasticizer	2.17

### Tests and analysis

The mechanical properties of nanocomposites and pullout strength were determined using a SANTAM STM150 Universal machine. The tensile, flexural and compressive tests were performed at loading rates of 5, 2 and 1.3 mm/min based on ASTM D638, D790 and D695 test methods, respectively. Three specimens were tested for evaluating each mechanical property. The pullout test was performed under tension mode based on ASTM C900 test method. The adhered steel rebar-concrete sample was placed in a steel fixture as the rebar were kept using the upside grip and the fixture was kept constant using the downside grip. The test was performed at tension rate of 10 mm/min. the steel rebar would exit gradually from concrete during loading (see Sect. 3S in supporting information). Three specimens were tested for evaluating pullout adhesion of each sample.

The grafting efficiency of raw and modified fumed silica were determined by thermogravimetric analysis (TGA) (Mettler-Toledo Co., Switzerland). FTIR was done by a TENSOR 27 to identify the chemical structure of nanoparticles and GPTMS. XPS was performed by ESCALAB 250 system (ESЄA System, USA). The particle size and morphology of the raw and modified fumed silica were examined by Field Emission Scanning Electron Microscopy (FE-SEM) (Hitachi, Japan). The X-ray diffraction (XRD) of the raw and modified fumed silica was determined by Bruker AXS diffractometer D8 (Madison, Wisconsin).

Dynamic mechanical thermal analysis (DMTA) was used to investigate viscoelastic behavior of nanocomposite epoxies (Netzsch Co., Germany). The water contact angle (WCA) of the fumed silica was measured using (UI-1220LE-M-GL, IDS-Germany).

## Results and discussions

### Finding the optimal pH for silanization step

**Figure 1:**
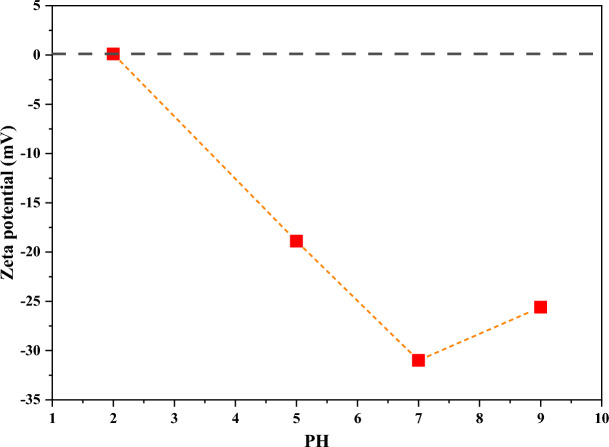
Zeta potential for nano silica in different acidic conditions.

The particles surface charge at different pH was investigated by evaluation of Zeta potential. The more negative zeta potential indicates more negative charge on the nanoparticle surface, better stability and better dispersion in the aqueous solvent. On this basis, samples with different pH of 2, 5, 7 and 9 were prepared and their zeta potential was measured (see Fig. 1).

The results indicated that at pH 7, the most negative charge of nano silica is achieved. This confirms the presence of a number of OH groups on the fumed silica surface which are ready to be silanized. On this basis, this pH was selected as the best condition for silanization step.

### Characteristics of the nanoparticles

**Figure 2:**
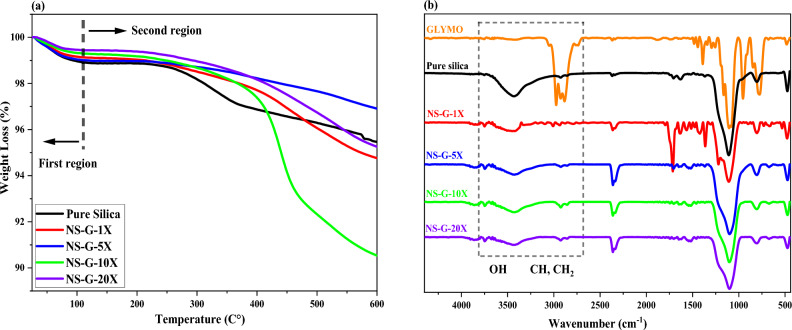
(**a**) TGA curves of raw silica and silanized silica, (**b**) FT-IR spectroscopy of GPTMS, raw silica and silanized silica.

Figure 2a shows the TGA results for the silanized and raw silica. As shown in the figure, all the samples exhibit large weight loss in the first region, which was related to the removal of the adsorbed H_2_O. The weight loss of modified nanoparticles were greater than pure nano silica in this region because of more water absorption during silanization process. In the second region, all the modified nanoparticles presented large weight losses, which were related to the degradation of grafted GPTMS. The weight loss of raw silica in this region corresponded to the de-hydroxylation of the OH groups. The highest weight loss in this region was obtained for the silanized silica (i.e. NS-G-10X sample). On this basis, this sample was selected as the optimal silanized for future processes. Figure 2b exhibits FTIR spectrums of the raw and silanized silica nanoparticles. In the spectrum of raw silica, the peaks at 961, 1528 and 1630 cm^−1^ were attributed to the bending vibration of O–H. The broad peak around 3450 cm^−1^ was related to the stretching vibration of O–H group. On this basis, there are three different types of hydroxyl groups on the surface of nanoparticles including covalently bonded, physically adsorbed and double-base hydroxyl groups.

For modified silica nanoparticles, the bending vibration peaks of OH group at 1630 cm^−1^ were disappeared and their peaks at 3450 cm^−1^ were weakened. This was attributed to consumption of surface OH groups during hydrolysis of GPTMS. Furthermore, the peaks at 1109, 805 and 475 cm^−1^ were related to asymmetric, symmetric and bending vibrations of Si–O–Si, respectively. The peaks at 2800–3000 cm^−1^ in the spectrum of GPTMS were related to stretching vibrations of the CH and CH_2_ groups. These peaks appeared again in the modified nanoparticles that confirm successful grafting of GPTMS molecules. In contrary, they were not seen in the spectrum of raw nanoparticle due to its inorganic nature. The peaks were intensified in the spectrum of modified nanoparticles at concentration of 10X that confirms higher GPTMS grafting ratio for this sample. The results were in good correlation with TGA findings. For convenience, the nanoparticles that modified at concentration of 10X is called modified nano silica (i.e. MNS) instead of NS-G-10X from now on. Table 4 shows the results of TGA.

**Table 4:** The results of Fig. 2.

Sample	Weight loss at 25–110 °C (%)	Weight loss at 110–800 °C (%)
NS	1.52	3.44
NS-G-X	0.86	4.37
NS-G-5X	1.02	2.12
NS-G-10X	0.71	8.78
NS-G-15X	0.53	4.16

**Figure 3:**
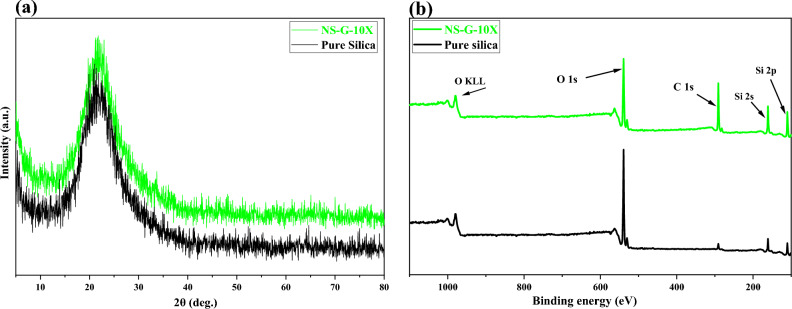
Characterization of raw and modified silica fume; (**a**) XRD and (**b**) XPS analysis.

XRD analysis was used to detect crystalline phase of the raw and modified nanoparticles (see Fig. 3). It is evident that the position of peaks has not been moved but the broad peak located at 22° shows that all the nanoparticles have amorphous structure. It was found that the surface modification of nanoparticles have not influenced their crystalline phase. The size of the crystals was also calculated according to the Scherrer equation^48,50,51^ that were 17.01 and 16.97 nm for raw and silanized silica. This result illustrates negligible reduction in the size of crystals after modification with GPTMS.

The XPS spectrums of the raw and surface optimal silanized silica are shown in Fig. 3. Narrow-scan spectra of C1*s*, O1*s*, and Si2*p* were used to determine changes in the chemical environment of the surface elements. The results confirmed successful grafting of GPTMS on the nanoparticles surface due to the intensified indicator peaks. Moreover, the coexisting peaks of Si2*p* (102.8 eV) and Si2*s* (156.3 eV) in the pure sample were related to the silanol group in silica structure. However, in the modified sample these peaks intensified due to the condensation of silane molecules that formed silanol layer on the nanoparticles surface. The XPS data are listed in Table 5.

**Table 5:** Results of XPS.

Peak	Binding energy (eV)	Composition (%)	
		Raw silica	NS-G-10X
C1s	281–287	4.26	30.01
O1s	530–536	71.33	38.23
Si2s	102–105	13.42	16.04
Si2p	99–101	10.99	15.72

**Figure 4:**
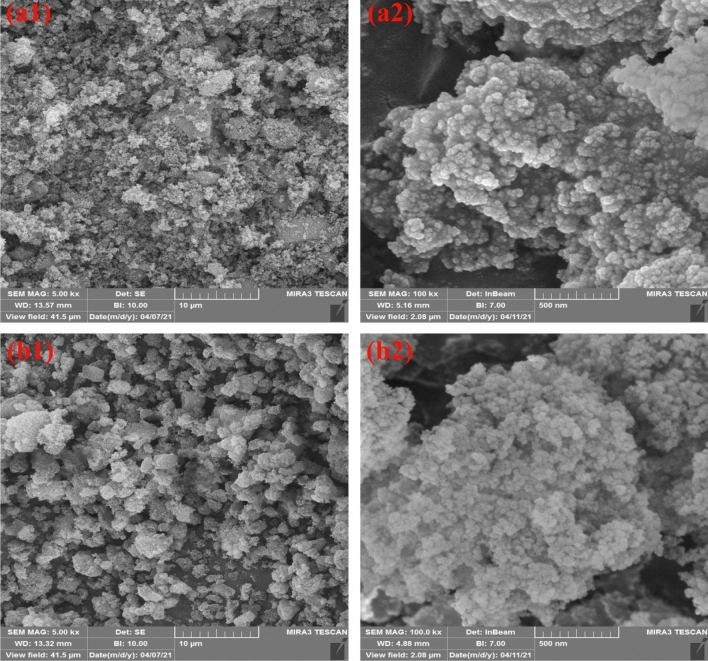
FE-SEM photography of; (**a**1, **a**2) raw silica and (**b**1, **b**2) silanized nanoparticles.

Figure 4 shows FE-SEM photography of raw and silanized silica. The pure SiO_2_ nanoparticles had sizes ranging from 25 to 30 nm. The agglomeration tendency of pure nano filler could be observed due to hydroxyl bonding between surface OH groups. After surface treatment of the silica with epoxy based GPTMS, the particle size reduced to 20–25 nm due to steric stabilization of grafted silanes.

### Mechanical performance of adhesives

**Figure 5:**
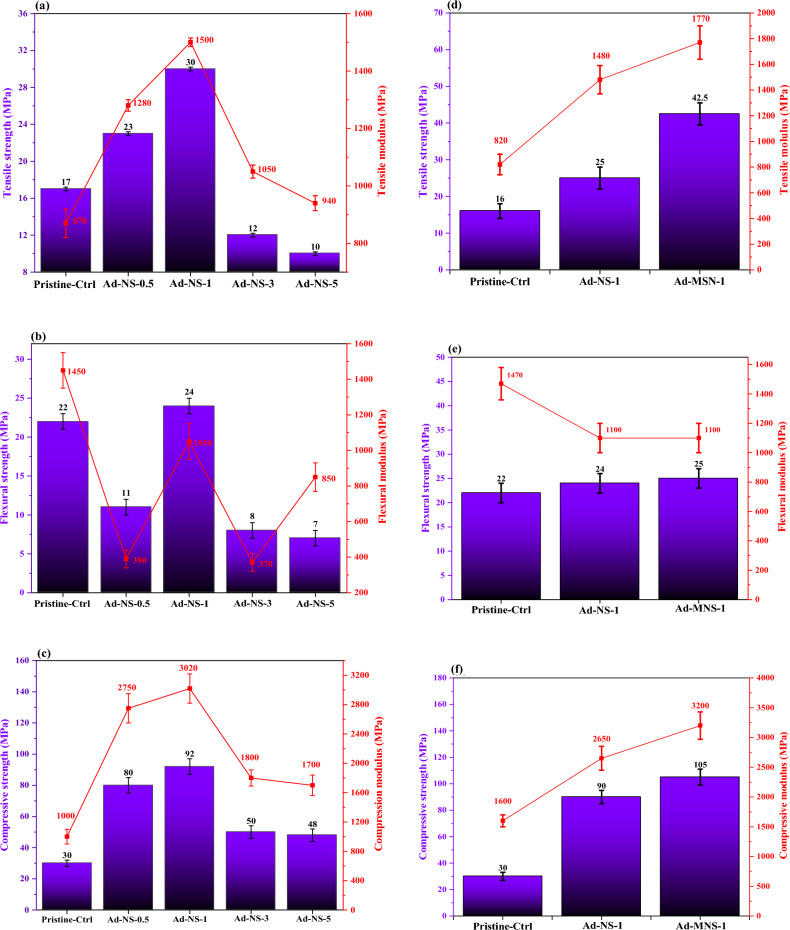
(**a**) Tensile, (**b**) flexural and (**c**) Strength and modulus properties of the pure nano silica and (**d**) tensile, (**e**) flexural and (**f**) compressive properties of the modified nano silica contained epoxy nanocomposites at different percentages of nanoparticles.

The raw silica was added to the resin part at four different concentrations. The nanocomposites were molded to prepare tests specimens. After curing, the specimens subjected to tensile, flexural and compressive tests. Figure 5 shows the results.

Based on the results, samples that contained 1wt% of raw silica showed the highest tensile, flexural and compressive properties among the other nanocomposites. It was concluded that at higher loading contents of the pure nano filler, the agglomeration of particles occurred that caused decrement in the mechanical properties. This confirms results of FE-SEM analysis.

The modified nanoparticles was also added to the epoxy resin at 1 wt%. The results were also shown in Fig. 5. It was seen that the tensile properties increased. It was attributed to the better dispersion of the silanized silica fume in the epoxy matrix due to steric stabilization of the nanoparticles that prepared by hindrance of the grafted silane groups^47,48^. The silanized silica fume showed no effects on the flexural properties but caused considerably increment in the strength and modulus properties of epoxy sample. The results were in good correlation to the results of FE-SEM and XRD analysis.

**Figure 6:**
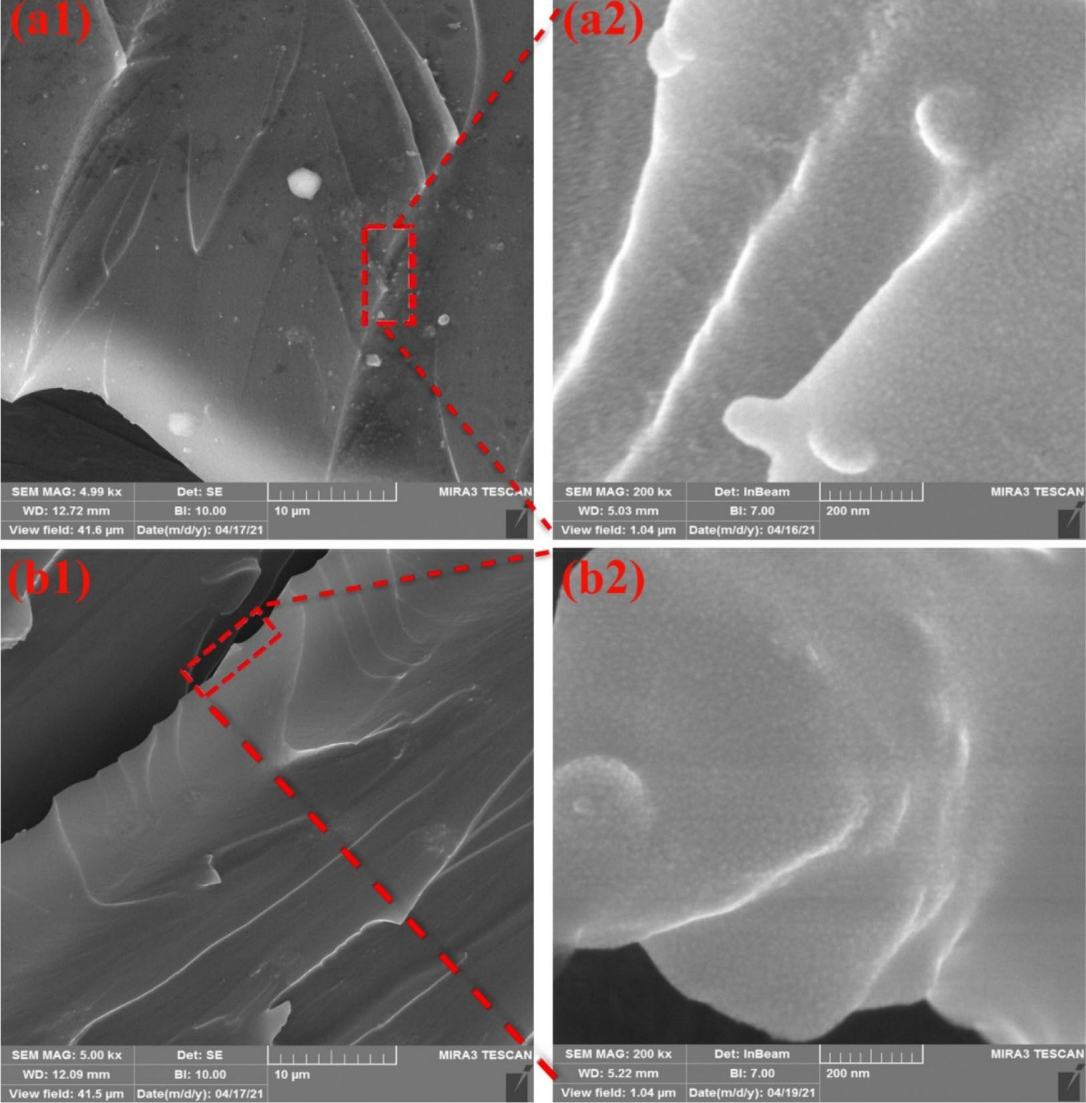
FE-SEM photography of; (**a**1, **a**2) raw and (**b**1, **b**2) modified epoxy nanocomposites.

**Figure 7:**
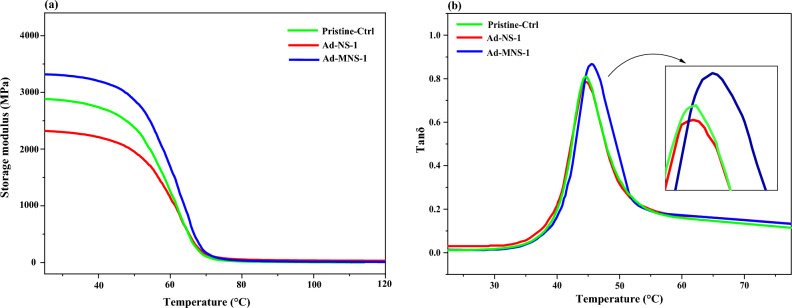
Mechanical properties of nanocomposites; (**a**) Storage modulus and (**b**) Tan δ.

Figure 6 shows FE-SEM images prepared from fractured surface of the nanocomposites at two magnifications (i.e. 10 µm and 500 nm). Big aggregates (i.e. with size of 100 nm–2.5 µm) was seen in the raw silica containing sample (see Fig. 7a). This was attributed to the hydrophilic nature of the silica fume that caused aggregation in the epoxy matrix. This indicates poor dispersion of hydrophilic silica fume in the epoxy matrix. After surface treatment of silica fume, considerable homogeneous dispersion and small-size aggregates (of up to 100 nm) were seen. This was attributed to the steric stability of the silica fume that was performed by grafted GPTMS. Besides, the river like lines increased in the fractured surface of modified nanocomposite that confirmed improved interactions between epoxy matrix and silanized nanoparticles^37,46,48^.

The dynamic-mechanical behavior of the nanocomposites and its relationship to the nanoparticle surface chemistry was investigated by DMTA analysis. Figure 7a shows the storage module curve in terms of temperature. The results showed that silanization of nanoparticles led to an increase in the storage modulus of the nanocomposite^52^. This should be attributed to the improved interfacial interactions between nanoparticles and epoxy matrix. This limits movement of the chains and increases stiffness of the matrix. The glass–rubber transition temperature (Tg) of the sample (derived from peak temperature of tanδ curve) showed slight increment that confirmed improved networking in the matrix due to attachment of nanoparticles to epoxy matrix (see Fig. 7b and Table 6)^53^.

**Table 6:** Tanδ max and Tg of the Resin samples.

Samples	Tanδ max	Tg at peak temperature (°C)
Pristine-Ctrl	0.81	55.6
Ad-NS-1	0.78	55.7
Ad-MNS-1	0.86	54.1

It was also observed that the pure nanoparticles caused decline in the storage modulus of the epoxy polymer which confirmed weak interfacial interactions between the polymer matrix and pristine nanoparticles. This was related to the weak filler–polymer interfacial interactions^47,53^. However, it had no effect on the Tg and tan δ of the cured pristine sample. This was attributed to the high crosslink density of the cured resin and its brittle behavior.

**Figure 8:**
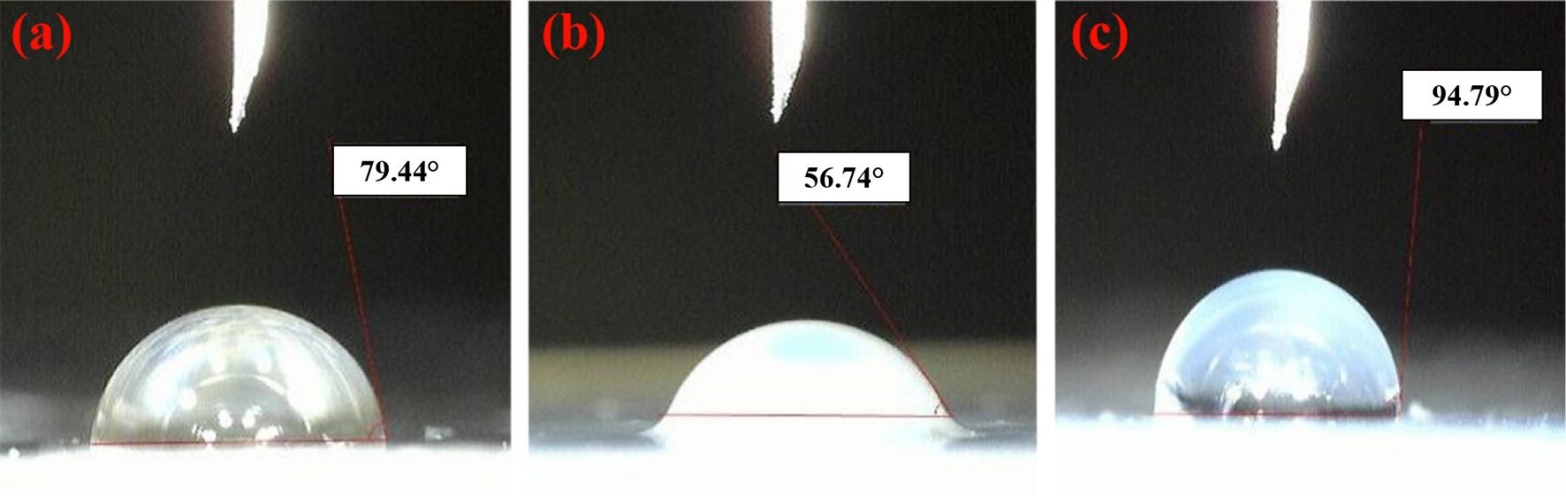
Water contact angle of (**a**) pristine epoxy, (**b**) raw silica containing epoxy nanocomposite (1 wt%) and (**c**) modified nano silica containing epoxy nanocomposite (1 wt%).

The WCA of the pristine epoxy and nanocomposite samples was measured that results are shown in Fig. 8. It is clearly seen that inclusion of pure nano silica caused increase in the hydrophilicity of the epoxy sample. In contrary, the modified nanoparticles increased hydrophobicity of the epoxy nanocomposite even more than pristine epoxy. This was attributed to presence of propyl and ethyl groups in the GPTMS structure that counteracted the effect of hydrophilic epoxide groups.

### Adhesion properties

**Figure 9:**
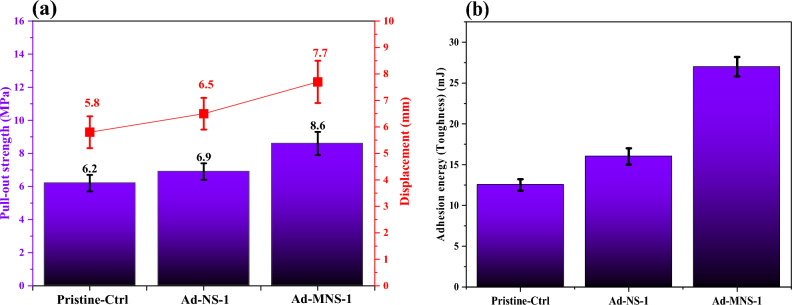
Pull-out properties of anchoring systems; (**a**) strength/displacement and (**b**) pullout energy.

Figure 9 shows the pull-out test results for the anchored steel rebar in concrete specimens by pristine epoxy adhesive and nanocomposite adhesives. The results indicated that by incorporating silanized silica fume to the epoxy based adhesives, the pull-out strength and displacement (i.e. toughness) incredibly increased. This was corresponded to the good interactions between modified silica fume with epoxy chains that prepared a nanocomposite adhesive with homogeneous dispersion of nanoparticles. This led the nanoparticles to diffuse into the micro/nano pores and cracks of concrete and increase in the steel rebar-concrete mechanical bonding. This also was related to the high toughness of the modified nanocomposite that increased its capability for damping the stresses.

In the case of using fumed silica in epoxy resin, no influences were seen in the adhesion properties. This was related to two different phenomena that occurred in the sample which counteracted the effects. In one hand, silica fume improved the mechanical properties of epoxy adhesive. In the other hand, the agglomerated nanoparticles at the interface of epoxy resin and concrete/steel surfaces decreased their effective contact area that could cause creation of micro cracks and fracture under pullout stress.

Figure 4S in supporting information shows comparative pullout load–displacement curves of the adhesives. It is clearly seen that rebar pulled out completely from the hole. The highest end displacement content is less than 8 mm.

Figure 9b shows the adhesion energy/toughness of the adhesives (i.e. based on the measured area under pullout load–displacement curves). It is obvious that pure silica nanoparticles improved toughness of the pristine adhesive layer but modified nanoparticles enhanced it incredibly.

**Figure 10:**
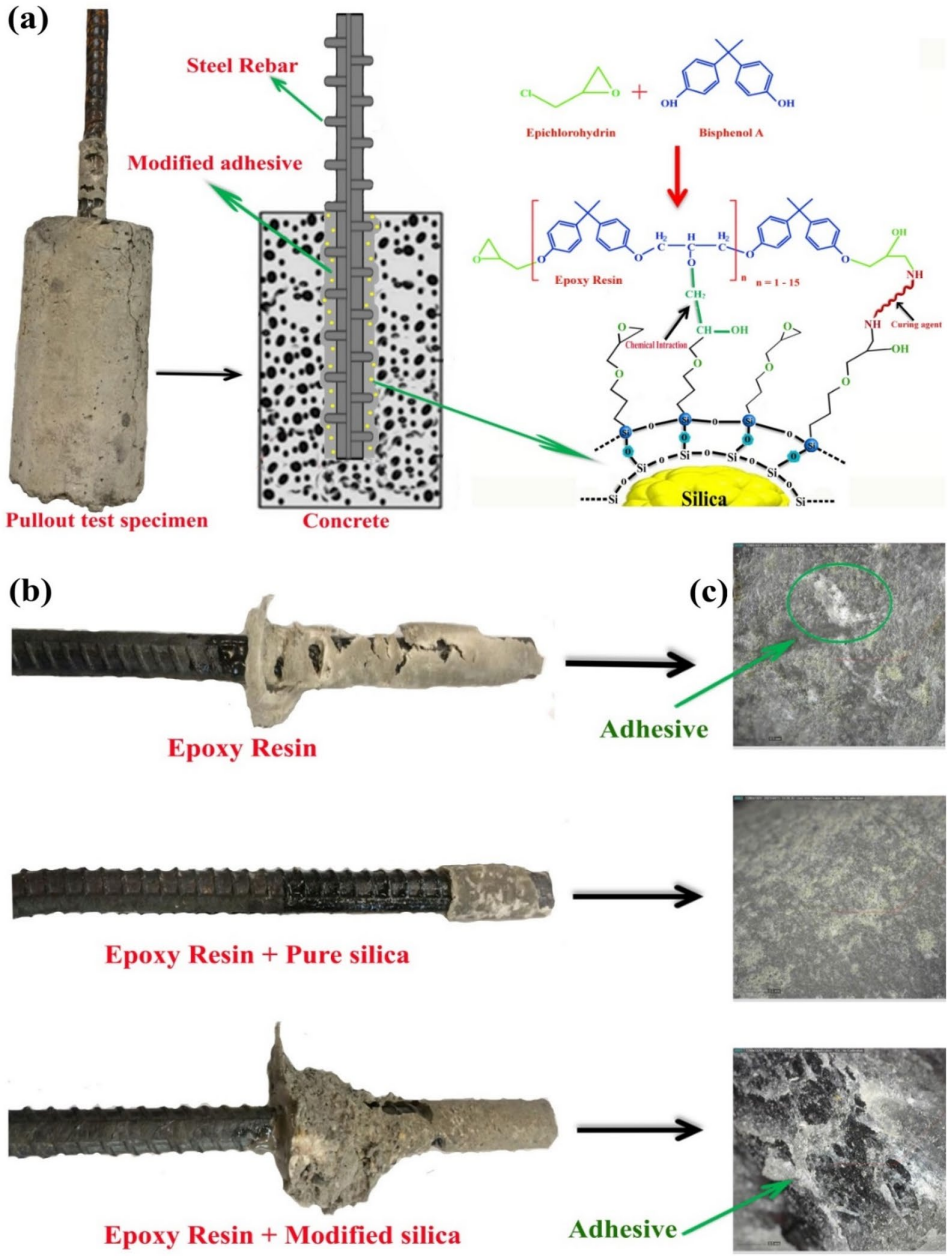
(**a**) Schematic of bonding mechanism of modified nanoparticles to epoxy resin via co-curing process, (**b**) images from surface of pulled out steel rebar from concrete for different samples, (**c**) surface images from concrete surface separated from steel rebar.

The significant increment in the bonding strength of modified nanocomposite adhesive to concrete/steel rebar surfaces should be corresponded to its different bonding mechanism. The present epoxide groups on the surface of silanized silica fume could react directly to amine groups of the curing agent. They also could react to the OH groups that created by epoxide rings opening through curing reactions. These covalent bonds increase the toughness and strength of the adhesive (see Fig. 10a). The hydrophobic nature of the modified adhesive prohibits accumulation of water at the interface of concrete and adhesive that usually happens during moisture exchange of hydrophilic concrete and decreases its interfacial interactions^54^.

On this basis, it could be claimed that the modified adhesive could decrease the corrosion rate of the steel rebar due to resistance against diffusion of water (i.e. as steel corrosive agent). Figure 10b exhibits images from the pulled out steel rebar surface. It is evidently seen that pristine adhesive showed cohesion defect in concrete phase. This means that the strength of concrete bulk is lower than adhesion strength of rebar-adhesive-concrete system^55^.

In contrary, by using pure nano silica in epoxy adhesive, the cohesion defect transferred to the adhesive layer that indicates lower strength of the adhesive. The modified nanoparticles improved adhesive interfacial interactions to the steel rebar and concrete. It also strengthened the adjacent concrete layer (i.e. due to diffusion into the surface crack and pores of concrete that increased volume of the adhered concrete to the steel rebar after pullout test).

Figure 10c shows few pristine adhesive patches on the surface of concrete. This means that it had better adhesion to the steel surface than concrete. In contrast, in the case of using silica fume containing adhesive, much more adhesive patches was seen on the concrete surface that means cohesion fracture of the adhesive layer due to its weakness. In the case of using modified adhesive, massive groves appeared at the concrete surface that created due to improved interfacial adhesion and cohesion fracture in the concrete bulk instead of adhesive layer.

## Conclusions

In this work, nano SiO_2_-epoxy adhesives were prepared for anchoring of steel rebar in concrete. The nanoparticles were firstly surface modified using an epoxy based silane to enhance their interfacial interactions to epoxy binder. The pullout adhesion test was performed for evaluation of adhesives performances. Based on the results the following conclusions were obtained:

- It was found that the highest silane grafting content was achieved at silane concentration of 10X (i.e. 10 times to stoichiometric concentration) and the surface modification had no effect on the crystalline phase of nanoparticles but decreased their sizes.
- Surface modification of nano silica caused increment in the WCA of epoxy film about 19 and 67% compared to the pristine epoxy and pure nanoparticle containing epoxy adhesives, respectively. It also changed the nature of the modified nanocomposite adhesive from hydrophilic to hydrophobic.
- Addition of the pure nano silica (1 wt%) to epoxy matrix caused 56, 81, 200 and 66% increment in the tensile strength, tensile modulus, compressive strength and compressive modulus improved by 56, 81, 200 and 66%, respectively.
- Incorporating 1 wt% of modified nano silica to epoxy adhesive caused 70, 20, 17 and 21% increment in the tensile strength, tensile modulus, compressive strength and compressive modulus compared to the epoxy adhesive filled by pure nano silica.
- Modified nano silica caused 16 and 43% increment in the storage modulus of the epoxy nanocomposite compared to the pristine epoxy and pure nano silica containing adhesives, respectively. It also increased 6 and 10% the loss function (tanδ), respectively.
- Using surface modified nano silica in epoxy matrix caused about 40 and 25% improvement in the pullout strength and 33 and 18% increment in the pullout displacement compared to the pristine epoxy and pure nano silica containing adhesives. The adhesion energy was enhanced up to 50 and 130% compared to the pure epoxy nanocomposite and pristine epoxy adhesives.

## Supplementary Information

Supplementary Information.

## Data Availability

It is confirmed that all Data Availability. The raw/processed data required to reproduce these findings can be shared.
