# Supplementary material for: Achieving outstanding mechanical/bonding performances by epoxy nanocomposite as concrete–steel rebar adhesive using silane modification of nano SiO2
Source: Sci Rep. 2023 Jun 6;13:9157. doi: 10.1038/s41598-023-36462-0 (PMC10244333; doi:10.1038/s41598-023-36462-0)
Supplement: Supplementary file 1 — Supplementary Information. [file 41598_2023_36462_MOESM1_ESM.docx]

**Supporting Information**

**Reza Ghamarpoor, Masoud Jamshidi*, Majid Mohammadpour**

*Constructional Polymers and Composites Research Lab., School of Chemical, Petroleum and gas Engineering, Iran University of Science and Technology (IUST), Tehran, Iran*

^*^ Corresponding author: Dr. Masoud Jamshidi, Associate professor, E-mail address: mjamshidi@iust.ac.ir, Tel. No.: ++98- 21-77240255, Fax No.: ++98-21-77240495.

**1S. Measuring the OH number of nano silica**

The amount of hydroxyl groups was obtained according to the following formula: [1, 2]:

$n_{OH}= N_{OH} \times\frac{1}{S_{BET}} = \frac{2[WL(T0)-WL(Tfinal)]}{M_{H2O}WL(Tfinal)} \times\frac{N_{A}}{S_{BET}} (\frac{hydroxyl}{{nm}^{2}})$ (1S)

**Table. 1S.** Information related to BET analyzes.

| Sample | Surface area (m^2^/g) | Mean pore diameter (nm) | Total pore volume (cm^3^ g^-1^) |
| --- | --- | --- | --- |
| raw Silica | **301.21** | **36.26** | **3.1** |

**Fig 1S.** Thermogravimetric analysis of the pure silica_._

Fig 1S shows the TGA of the raw silica. TGA and BET analysis were used to calculate the amount of OH groups. The weight loss difference of the samples between 115 and 410 °C was related to the OH groups. The measured weight loss was about 3.1% for raw silica fume.

Using equations 1, the number of surface OH groups, the mole content of surface OH groups and stoichiometric content of GLYMO (X) was obtained for 0.5 gram of raw silica fume as following:

$N_{OH}=16.725\times{10}^{20}$ (2S)

$n_{OH}= N_{OH} \times\frac{1}{S_{BET}} = 10.4672 (\frac{hydroxyl}{{nm}^{2}})$ (3S)

$m_{EPPTMS}=0.657 gr$ (4S)

**2S. Preparing pullout test specimens**

| __  **(a)** | __  **(b)** |
| --- | --- |

**Fig 2S.** a) The cylindrical mold with a rod in the center and (b) the molded fresh concrete.

**3S. Pullout test procedure**

Figure 3S shows the pullout test setup.

**The pullout test specimen**

**The Upside grip**

**The fixture of test specimen**

**The end tail of embedded steel rebar in concrete**

**Fig. 3S.** The pullout test.

Figure 4S shows an obtained pullout load-displacement curve.

**Fig. 4S.** The pullout load-displacement curve.

1. Mrkoci MI. Influence of silica surface characteristics on elastomer reinforcement: Queen's University; 2001.

2. Ek S, Root A, Peussa M, Niinistö L. Determination of the hydroxyl group content in silica by thermogravimetry and a comparison with 1H MAS NMR results. Thermochimica acta. 2001;379(1-2):201-12.
